# IDENTITY AND RESPONSIBILITY: WHAT IT MEANS TO BE A CAREGIVER AND ITS IMPLICATIONS FOR POLICY AND HEALTH CARE

**DOI:** 10.1093/geroni/igz038.497

**Published:** 2019-11-08

**Authors:** Minakshi Raj, Jodyn Platt, Tom Fitzgerald, Denise Anthony, Shoou-Yih Daniel Lee

**Affiliations:** 1 University of Michigan School of Public Health, Ann Arbor, Michigan, United States; 2 University of Michigan Medical School, Ann Arbor, Michigan, United States

## Abstract

Improving health care and quality of life for older adults in the U.S. requires increased attention to the informal caregivers supporting aging relatives. Studies estimate that over 40 million unpaid caregivers provide more than $400 billion of unpaid care; however, there is little research examining the scope and variety of support caregivers provide. Less research has examined how caregivers conceptualize their experience beyond “caregiver burden”. A more comprehensive understanding of the types of support caregivers provide and caregiver perceptions of their role and purpose are critical to enhancing policies, e.g., the Family and Medical Leave Act, to be more responsive to caregiver needs. This study seeks to (a) identify the ways informal caregivers provide support and (b) describe what it means to be a caregiver from the perspective of informal caregivers. We conducted four focus groups with informal caregivers (relatives or partners) in Southeastern Michigan (n=18) and conducted qualitative thematic analyses. Support that caregivers provide ranges from financial planning to medical decision-making to social engagement. While caregivers face frustration and isolation consistent with “caregiver burden,” they also recognize positive attributes of being a caregiver such as overcoming adversity and helping elderly relatives maintain dignity. These attributes contribute to their identity of what it means to be a caregiver and perceived capacity to improve their relative’s health. Our findings suggest that we are underestimating the scope of work involved in care-giving and that a wider range of activities should be considered when developing workplace policies and resources to promote caregiver wellness.

